# Nanoparticle-Enhanced Acoustic Wave Biosensor Detection of *Pseudomonas aeruginosa* in Food

**DOI:** 10.3390/bios15030146

**Published:** 2025-02-25

**Authors:** Sandro Spagnolo, Katharina Davoudian, Brian De La Franier, Robert Kocsis, Tibor Hianik, Michael Thompson

**Affiliations:** 1 Faculty of Mathematics, Physics and Informatics, Comenius University, Mlynská dolina F1, 84248 Bratislava, Slovakia; sandrospagnolo1@gmail.com (S.S.); tibor.hianik@fmph.uniba.sk (T.H.); 2 Department of Chemistry, University of Toronto, 80 St. George Street, Toronto, ON M5S 3H6, Canada; k.davoudian@mail.utoronto.ca (K.D.); brian.delafranier@mail.utoronto.ca (B.D.L.F.); 3 Hungarian Dairy Research Institute Ltd., 1 József Csiszár Street, 9200 Mosonmagyaróvár, Hungary; rkocsis@mtki.hu

**Keywords:** antifouling linker, DNA aptamers, aptasensor, *Pseudomonas aeruginosa*, thickness shear mode, dissipation, gold nanoparticles

## Abstract

A biosensor was designed for detecting *Pseudomonas aeruginosa* (*P. aeruginosa*) bacteria in whole milk samples. The sensing layer involved the antifouling linking molecule 3-(2-mercaptoethanoxy)propanoic acid (HS-MEG-COOH), which was covalently linked to an aptamer for binding *P. aeruginosa*. The aptasensor uses the thickness shear mode (TSM) system for mass-sensitive acoustic sensing of the bacterium. High concentrations (10^5^ CFU mL^−1^) of nonspecific bacteria, *E. coli*, *S. aureus*, and *L. acidophilus*, were tested with the aptasensor and caused negligible frequency shifts compared to *P. aeruginosa*. The aptasensor has high selectivity for *P. aeruginosa*, with an extrapolated limit of detection (LOD) of 86 CFU mL^−1^ in phosphate-buffered saline (PBS) and 157 CFU mL^−1^ in milk. To improve the sensitivity of the sensor, gold nanoparticles (AuNPs) were functionalized with the same aptamer for *P. aeruginosa* and flowed through the sensor following bacteria, reducing the extrapolated LOD to 68 CFU mL^−1^ in PBS and 46 CFU mL^−1^ in milk. The frequency variations in the aptasensor are proportional to various concentrations of *P. aeruginosa* (10^2^–10^5^ CFU mL^−1^) with and without AuNPs, respectively. The low and rapid mass-sensitive detection demonstrates the ability of the aptasensor to quantitatively identify bacterial contamination in buffer and milk.

## 1. Introduction

The quality control of milk is crucial for public health, since pathogenic microorganisms can contaminate milk at any step of its processing. Detecting and quantifying pathogens in milk is important for ensuring product safety and that concentrations do not surpass regulated limits [1]. At present, analyzing bacteria depends on the time-consuming procedure of growing bacteria on agar plates and counting colonies the following day [2]. Biosensors offer a promising alternative, since the ideal method for detection should be rapid, simple to operate, and not depend on specialized personnel.

Various bacteria can contaminate milk, such as *Pseudomonas aeruginosa* (*P. aeruginosa*). The pathogen is particularly problematic, since it is a psychrotrophic bacterium that is capable of growing its population and producing heat-resistant enzymes at cool temperatures, even below temperatures of refrigeration [3]. Just 1 CFU mL^−^^1^ of psychrotrophic bacteria may be sufficient to cause milk spoilage within a five-day period [4]. To our knowledge, there is, therefore, no acceptable limit for *P. aeruginosa* and other psychrotrophic bacteria in milk. Although pasteurization can kill *P. aeruginosa*, its heat-stable enzymes can degrade milk components, leading to spoilage. Proteases and lipases have the most significant effect on milk products, decreasing the shelf life and nutritional value of these products [3]. Without pasteurization, exposure to milk contaminated with *P. aeruginosa* can lead to dangerous infections that can cause deadly conditions such as pneumonia or sepsis. Pseudomonas infections are particularly hazardous since the bacterium can have resistance to many antibiotics [2].

In addition to milk, *P. aeruginosa* can colonize many environments, especially those with high humidity. These Gram-negative bacteria can be found in places that handle food and beverages, leading to the possibility for contamination. *P. aeruginosa* is commonly present in raw vegetables and dairy products due to encountering contaminated water. The regulated maximum concentration of *P. aeruginosa* in drinking water is 3 CFU L^−^^1^ in Canada, United States, Europe, Japan, Brazil, and according to the World Health Organization (WHO) [1]. However, it is not always easy to detect this concentration.

Since *P. aeruginosa* can grow at cool temperatures, produce heat-resistant enzymes, and rapidly develop biofilms that can be challenging to remove, it is critical to detect the bacterium and its enzymes as quickly as possible [3]. Detecting directly in undiluted milk is ideal and requires antifouling strategies to prevent the unwanted non-specific adsorption of milk components (e.g., proteins and lipids) on the sensing surface. Fouling of such milk components can be mitigated by the covalent functionalization of antifouling layers on the surface of the sensor. One common method of antifouling is based on mono- or polyethylene glycol layers, such as the self-assembled monolayers of 3-(2-mercaptoethanoxy)propanoic acid (HS-MEG-COOH) or 2-(2-mercaptoethoxy)ethan-1-ol (HS-MEG-OH), which have polar functional groups that interact with water molecules in the milk and lead to the formation of a “water barrier” on the surface. This zone of hydration makes non-specific adsorption thermodynamically unfavourable [5].

Combining an antifouling sensing layer with a probe that is specific to *P. aeruginosa* allows the biosensor to detect the bacterium with high specificity in a complex medium like milk. In this work, an aptamer was used as a probe, which was selected through Cell SELEX for binding to *P. aeruginosa*. Cell-SELEX was designed to target whole cells to produce aptamers that, in our case, specifically recognize the surface of *P. aeruginosa* [6]. Since cell-SELEX uses the whole bacterium, the binding site of the aptamer to the cell is unknown. However, the specificity of the aptamer is determined by testing with other bacterial strains [7]. To detect *P. aeruginosa*, the aptamer probes were bonded via the carboxylic acid groups of a self-assembled monolayer of HS-MEG-COOH, which anchors the bacteria upon binding.

To increase the sensitivity of the aptasensor, gold nanoparticles (AuNPs) functionalized with the same aptamer were incorporated. There is growing interest in employing nanoparticles for biosensors since AuNPs can form an interface between biorecognition and transduction. The nanoparticles amplify the sensitivity of detection by interacting with the analyte and act as an enhancer. Since the AuNPs are simple to use, they facilitate a diverse application in the field of mass-sensitive biosensors for improved analytical performance. Amplification of sensitivity has been reported in other works, such as for the detection of Avian Influenza Virus with aptamer-coated magnetic nanobeads [8] or detecting thrombin with aptamer-functionalized AuNPs [9].

The *P. aeruginosa* aptasensor was designed on the gold electrode surface of thickness shear mode (TSM) discs. TSM analyzed the frequency shifts during surface functionalization and bacterial binding. The mass-sensitive sensor involves the application of an alternating voltage across the gold-plated quartz disc, causing the crystal to resonate. Any adsorption or loss of mass on the surface affects the resonance and can be detected as a frequency change (Δ*f*) [10]. To confirm the functionalization of the TSM disc surface, contact angle goniometry (CAG) provided a qualitative comparison of different self-assembled monolayers (SAM). Following bacterial measurements, the surfaces of the TSM discs were imaged with scanning electron microscopy to visualize the bound bacteria. The bound nanoparticles were imaged with transmission electron microscopy.

We describe the detection of *P. aeruginosa* with a piezoelectric acoustic sensor, which is a less common transduction method in the literature, particularly compared to electrochemical aptasensors. The developed sensor involves aptamers anchored on an antifouling surface, as well as increased biosensor sensitivity and therefore improved detection by incorporating gold nanoparticles (AuNPs). The antifouling aptasensor demonstrates the ability to rapidly and selectively detect bacteria directly in undiluted whole milk samples, which is crucial for efficient quality control in the dairy industry.

## 2. Materials and Methods

### 2.1. Materials

HS-MEG-OH and HS-MEG-COOH were synthesized based on previous procedures [5,11]. Anhydrous magnesium sulphate, sodium thiosulfate, sodium carbonate, sodium iodide, sodium chloride, and absolute ethanol were obtained from the University of Toronto (Toronto, ON, Canada). Other organic solvents (methanol, ethyl acetate, hexane, dichloromethane, and acetonitrile) were analytical grade and from Sigma–Aldrich (Darmstadt, Germany). T-butyl acrylate, hydrogen peroxide, zinc, trifluoroacetic acid, β-mercaptoethanol, N-hydroxysuccinimide (NHS), 1-(3-(dimethylamino)propyl)-3-ethylcarbodiimide hydrochloride (EDC), and ethanolamine were purchased from Sigma–Aldrich (Darmstadt, Germany). Chemicals were used without further purification. Ammonium hydroxide (NH_4_OH) and N-Benzyltrimethylammonium hydroxide (40% in methanol *w*/*w*) were purchased from Fisher Scientific (Ottawa, Canada). AuNPs (5 nm diameter, OD 1, stabilized suspension in 0.1 mM phosphate-buffered saline (PBS), reactant free) were purchased from Sigma–Aldrich (Darmstadt, Germany). Aqueous solutions were prepared using Milli-Q water (18.2 MΩ.cm).

The lyophilized aptamer against *P. aeruginosa* (5’-CCC CCG TTG CTT TCG CTT TTC CTT TCG CTT TTG TTC GTT TCG TCC CTG CTT CCT TTC TTG-3’, with a 3’ amino modification) was purchased from Generi Biotech (Hradec Králové, Czech Republic). The aptamer sequence follows these cited works [12,13,14,15,16,17,18]. A 100 μM stock solution of the lyophilized aptamer was prepared in DNase-free TE buffer (10 mM Tris-HCl, 1 mM EDTA, pH 8.0). This stock solution was divided into 100 μL aliquots and stored at −20 °C. Before functionalizing the gold electrodes with aptamer, an aliquot was diluted with Milli-Q water to a concentration of 5 μM. The secondary structure of the aptamer was analyzed with OligoAnalyzer Tool^TM^ software (Integrated DNA Technologies, Coralville, IO, USA) and the results are reported in the Supplementary Material (see Supplementary Material, Section S1).

PBS was used as a binding buffer and was prepared with 137 mM NaCl, 2.7 mM KCl, 10 mM Na_2_HPO_4_, and 1.8 mM KH_2_PO_4_ at pH 7.4. A 0.22 μm membrane (Merck-Millipore, Darmstadt, Germany) was used to filter every solution. *Pseudomonas aeruginosa* PAO1 was provided by the National Collection of Agricultural and Industrial Microorganisms (Budapest, Hungary) and *Lactococcus acidophilus* LA-5 was kindly supplied by C. Hansen (Hørsholm, Denmark). *Escherichia coli* DH5α and *Staphylococcus aureus* KR3 were purchased from the University of Toronto Medstore (Toronto, ON, Canada). Whole UHT cow’s milk (3.5% fat) was bought from LIDL (Bratislava, Slovakia) and from Walmart (Toronto, ON, Canada).

### 2.2. Surface Modification of TSM Crystals

**Figure 1:**
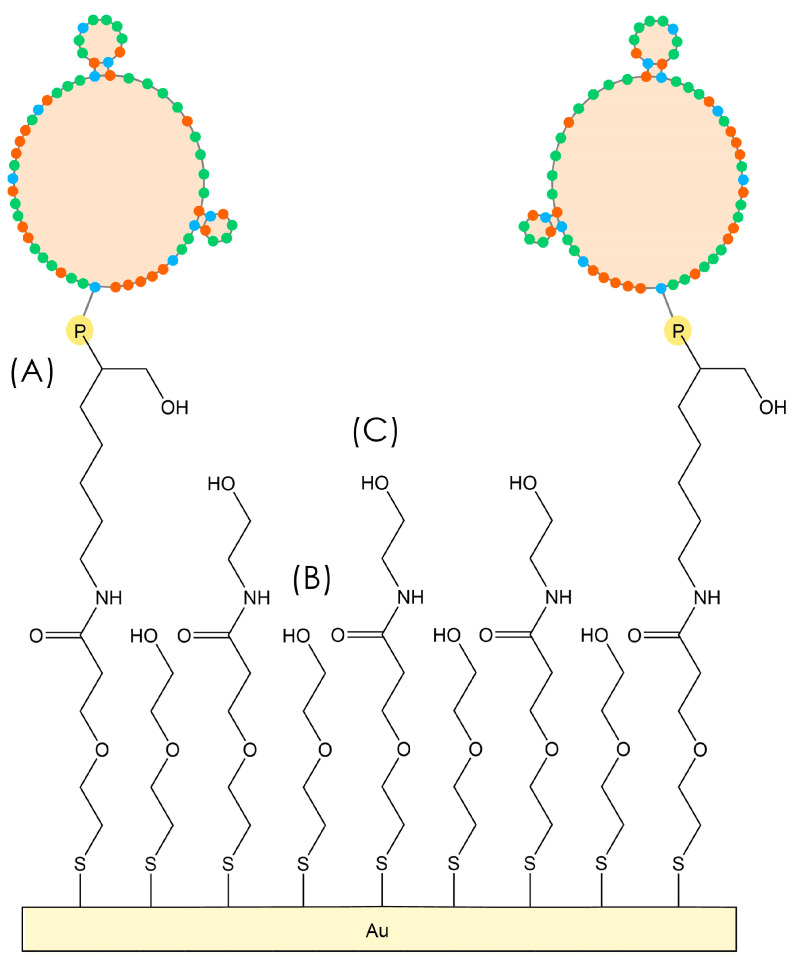
The functionalization of the TSM crystal to build the aptasensor, where (**A**) aptamers are covalently bonded to the HS-MEG-COOH linker, (**B**) HS-MEG-OH antifouling molecules act as spacers, and (**C**) HS-MEG-COOH molecules that did not bind to aptamers are, instead, elongated with ethanolamine. The coloured dots of the aptamers represent cytosine (orange), guanine (blue), and thymine (green) nucleic acids.

AT cut quartz crystals (8 MHz fundamental frequency, gold electrodes with a 0.2 cm^2^ area deposited on each side) were purchased from Total Frequency Control Ltd. (Storrington, UK). The cleaning of the gold sensor surfaces involved 2 mL of basic piranha solution (1:1:3 *v*/*v* 28–30% NH_4_OH, 30% H_2_O_2_, Milli-Q water at 75 °C) with three 30 min cycles. After each cycle of piranha cleaning, the crystals were rinsed with Milli-Q water three times. After the final cycle and Milli-Q rinse, the discs were rinsed twice with methanol, dried with a gentle flow of nitrogen, and stored in absolute ethanol or functionalized with the thiol solution. The functionalization was conducted by immersing the crystals in 0.5 mM HS-MEG-OH, 0.5 mM HS-MEG-COOH, or in a 1:1 *v*/*v* solution of 0.5 mM HS-MEG-OH and 0.5 mM HS-MEG-COOH (with a final concentration of 1 mM of thiol, labeled HS-MEG-Mix), in absolute ethanol overnight. After functionalization, the crystal surfaces were modified in vials. The carboxylic acid terminal groups of HS-MEG-COOH were first activated with 20 mM NHS and 50 mM EDC in Milli-Q water for 35 min, followed by incubation in a solution of the *P. aeruginosa* amino-aptamer (2 μM in Milli-Q water) for 90 min, and then a solution of 0.1 M ethanolamine in Milli-Q water for 45 min. Between the functionalization steps, the discs were rinsed with Milli-Q water and dried under a gentle flow of nitrogen. For some crystals, surface modification was performed in flow at 50 µL/min with the TSM instrument to monitor the resonance frequency changes and understand whether functionalization was successful. The scheme of the aptasensor surface is represented in Figure 1.

### 2.3. Contact Angle Goniometry Analysis

Cleaned or functionalized crystals were qualitatively compared using static contact angle goniometry. Measurements were conducted in triplicate with Milli-Q water (7 μL) at room temperature using the KSV CAM 101 goniometer (KSV Instruments Ltd., Helsinki, Finland).

### 2.4. Bacteria Preparation

*P. aeruginosa* was grown overnight in lysogeny broth at 37 °C. The bacterial solution was then serially diluted 1/10 to 1/10^9^-fold in PBS. Each solution was applied on agar plates (3 × 10 μL). The optical density at 600 nm (OD600) was measured using a UV-1600PC spectrometer (VWR International, Mississauga, ON, Canada). The plates were incubated overnight at 37 °C and the following day the colonies were counted to calculate CFU per OD600 (see Supplementary Material, Section S2). To analyze the specificity of the TSM sensor, we also used *S. aureus*, *E. coli*, and *L. acidophilus* bacteria. They were cultivated overnight at 37 °C, and subsequently 1.5 mL of bacterial solution was centrifuged at 14,500 RPM for 10 min, the supernatant was removed, and the bacteria pellet was resuspended in PBS. Before inserting the bacterial suspension into the sensor, the OD600 was measured to calculate the base concentration (CFU mL^−1^), then the solution was diluted with PBS to obtain the desired bacterial concentration. The relationship between the optical density and the cell concentration of different bacteria was obtained from previous works (for *P. aeruginosa*: 0.37 OD = 6 × 10^7^ CFU mL^−1^) [7,19,20,21].

### 2.5. Preparation of AuNPs

**Figure 2:**
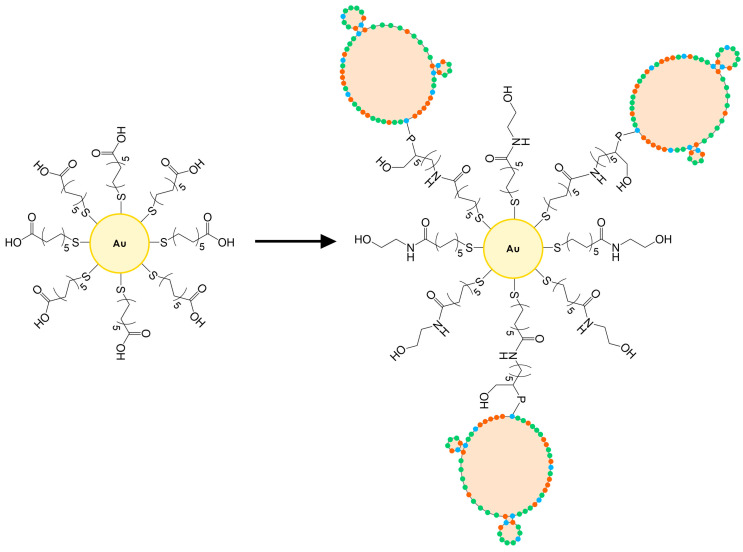
The scheme of the functionalization of AuNPs with DNA aptamers. MUA-functionalized AuNPs were modified with NHS/EDC, aptamer, and then ethanolamine. The number of bound aptamer molecules as well as the size of the AuNPs are only intended to describe the functionalization process and do not reflect the actual size. The coloured dots of the aptamers represent cytosine (orange), guanine (blue), and thymine (green) nucleic acids.

A stabilized suspension of 5 nm diameter AuNPs in PBS buffer with an OD of 1 (90.91 mM) was used. The maximum wavelength of absorbance and molar extinction coefficient of these nanoparticles were 515–520 nm and 1.10 × 10^7^ M^−1^ cm^−1^, respectively. The surface of the AuNPs was subsequently functionalized with the same mixed monolayer as those used for sensor preparation via ligand exchange in the following way: a stock solution of the mixed thiols was prepared at 10 mM concentration in PBS (11 mL) and it was used to dilute 2 mL of the stock of AuNPs to the final concentration of 14 mM of AuNPs. Functionalization with thiols was carried out overnight. Subsequently, the nanoparticles were precipitated by centrifugation at 14,500 RPM for 15 min and washed with PBS buffer three times. Finally, the nanoparticles were resuspended in the same initial volume with a solution of NHS/EDC (the same concentration used to functionalize the gold electrodes) and incubated for 55 min while stirring. Then, the solution was eliminated after precipitating the nanoparticles by the same centrifugation procedure, and three washes were performed with PBS. The nanoparticles were then resuspended with the same solution containing the aptamer used for the functionalization of the TSM electrodes. After incubating for 120 min, three washes were performed with PBS to remove unbound nucleic acid. Subsequently, the nanoparticles were resuspended with a 0.1 M ethanolamine solution in PBS and left to incubate for 60 min to passivate the still active carboxyl sites. The solution was removed by the same procedure and three final washes with PBS were performed. The functionalized nanoparticles were resuspended in PBS at a concentration to have an optical density of about 0.50 and stored at 4 °C prior to use (Figure 2). The aptamer-functionalized AuNPs were characterized with UV-Vis to identify the characteristic peaks of gold nanoparticles and DNA (see Supplementary Material, Section S3).

### 2.6. TSM Set-Up and Data Analysis

TSM crystals were inserted in an acrylic flow-through cell (Johannes Kepler University, Linz, Austria) [22], which was tightened with a holder and connected to a vector analyzer (SARK-110, Seeed, Shenzhen, China). The resonance frequency and resistance data were collected with a Python software (version 3.6.7) [23]. PBS was used as the running buffer, which was pulled by a GeniePlus pump (Kent Scientific, Torrington, CT, USA) with a flow of 50 μL/min. PBS buffer was flowed until the resonance frequency stabilized (about 50 min). After frequency stabilization, 250 μL of PBS or whole milk spiked with bacteria was flowed for 5 min, followed by PBS running buffer. The same measurements were repeated without bacteria as a control.

For in-flow functionalization of the crystal, the following solutions were used: HS-MEG-COOH solution (25 min), a PBS rinse, NHS/EDC (35 min), a PBS rinse, aptamer solution (90 min), a PBS rinse, ethanolamine solution (40 min), and PBS until frequency stabilization was reached. HS-MEG-COOH was used to passivate possible exposed regions on the gold surface and ethanolamine reacted with any remaining activated carboxylic acid groups not bound with aptamer. PBS rinses typically lasted for five minutes. After resonance stabilization, whole milk or PBS (with or without bacteria) was flowed for 5 min, followed by PBS until a stable baseline was reached. Measurements were made in triplicate.

A Python code based on the equation of Yoon et al. [24] was used to fit the data that were collected from the TSM measurements. Data analysis and statistical processing were conducted using OriginPro 8 (OriginLab Corporation, Northampton, MA, USA). 

### 2.7. TEM and SEM Microscopy Analysis

The nanoparticles were deposited onto TEM grids (Ultrathin Carbon Film on a Lacey Carbon Support Film, 400 mesh, Copper; Ted Pella Inc., Redding CA, USA). Images were taken on a High-Resolution Transmission Electron Microscope (JEOL JEM 2010 from JEOL Canada Inc., Saint-Hubert, QC, Canada) at an acceleration voltage of 100 kV. The size was calculated with ImageJ software, version 1.54d.

Following the measurements, the surface morphology of the crystals was analyzed with environmental scanning electron microscopy (FEI Quanta 250 FEG). The discs were fixed with a conductive paste to standard SEM holders. The samples were measured in high vacuum mode at 5 eV.

## 3. Results and Discussion

### 3.1. Contact Angle Goniometry

**Figure 3:**
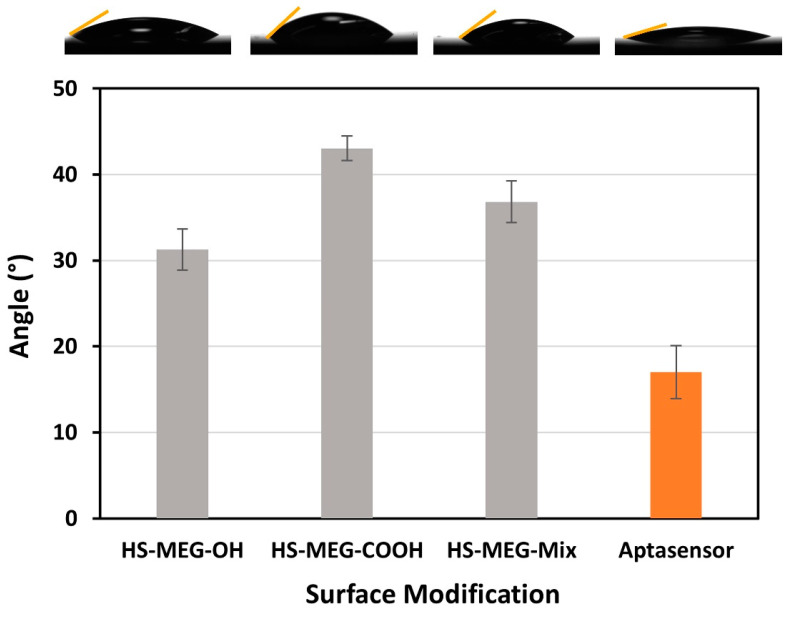
A comparison of the contact angles on layers on TSM crystals. Results from our previous work are shown in grey, compared to the aptasensor for *P. aeruginosa* designed in this work. The measurements were conducted in triplicate and the error bars represent the standard deviation.

The functionalized surfaces of the TSM discs were confirmed using contact angle goniometry (Figure 3). The analysis of new thiol layers (HS-MEG-COOH, HS-MEG-OH, and HS-MEG-Mix) were studied in previous works [4,25], while we focused on the aptasensor in this study. Compared to the contact angle on bare gold (about 56° ± 4°) [25], the thiol and the aptasensor surfaces have greater wettability. The contact angle of the aptasensor had the lowest average angle, about 17.0° ± 6.2°. The higher wettability of the aptasensor confirms the aptamer/ethanolamine coating that renders the surface more hydrophilic.

### 3.2. Milk Antifouling Test with TSM

**Figure 4:**
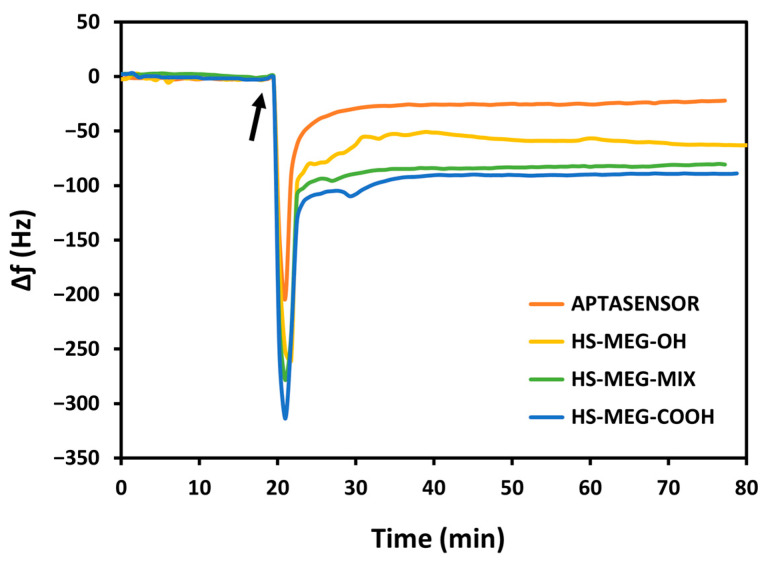
The decrease in frequency upon exposing milk to various functionalized surfaces on the TSM crystal. The arrow indicates the addition of whole milk on the sensing surface.

For the study of the antifouling properties of the synthesized thiols (HS-MEG-OH, HS-ME-COOH, HS-MEG-Mix) and of the coatings on the crystals, the surfaces were exposed to whole milk samples (Figure 4). Milk was chosen since it has a variety of substances, such as fats, carbohydrates, and high protein content. In this test, the difference in the antifouling properties of the layers obtained from the thiols and the aptasensor was investigated.

Table S4.1 in the Supplementary Material reports the average frequency shifts due to milk fouling on the modified surfaces of the crystals. The HS-MEG-COOH surface had the most fouling (92.0 ± 14.3 Hz) compared to HS-MEG-OH crystals (50.0 ± 15.0 Hz). Negatively charged carboxylic acids likely engage in more interactions with certain milk components, leading to increased fouling. In contrast, milk adsorption on the thiol mix (80.9 ± 0.6 Hz) was intermediate compared to discs functionalized with single thiols. These values confirm the presence of the thiol mix on the surface.

**Figure 5:**
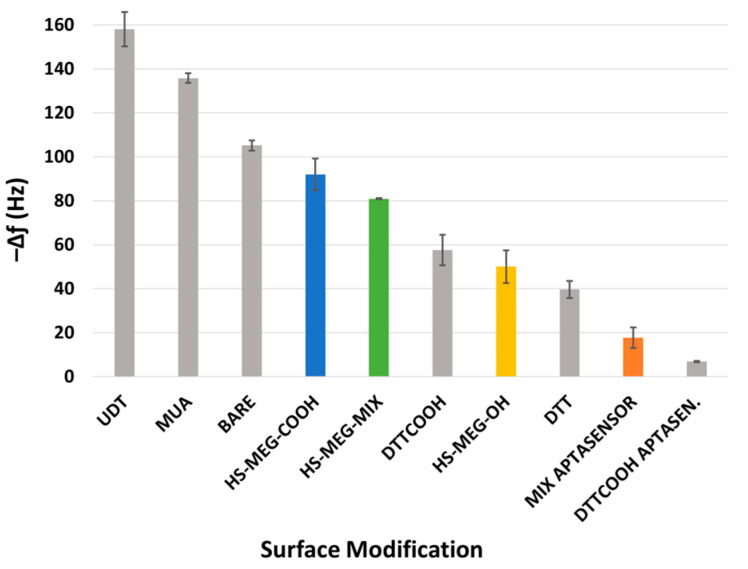
A comparison of the fouling of whole milk on the bare gold electrode and various surface modifications. The results of this study are shown in colour, while the results of the previous work are in grey [21].

Milk fouling on the aptasensor caused a significantly smaller frequency change (17.7 ± 9.4 Hz), indicating that the aptasensor has enhanced antifouling behavior. This can be interpreted considering the properties of the water barrier: the interactions between the water molecules and the inner areas of the layer may increase the thickness of the barrier itself. HS-MEG-COOH was elongated with either aptamer or ethanolamine, while HS-MEG-OH remained unbound. This created a surface with alternating longer (HS-MEG-COOH + aptamer/ethanolamine) and shorter (HS-MEG-OH) molecules, facilitating the formation of a more extensive water barrier as water molecules can penetrate deeper into the layer. A similar fouling phenomenon was observed in our recent work [21] for the new antifouling thiol molecule 3-dithiothreitol propanoic acid (DTT_COOH_), as shown in Figure 5. Incorporating the linker into a sensing layer with an aptamer bound to the carboxyl group removes the net surface charge and reduces fouling.

When comparing our previous work [21] with the current study (Figure 5), HS-MEG-COOH had more milk adsorption than DTT_COOH_, but less than non-antifouling thiols. HS-MEG-OH, already studied as an antifouling molecule [11], had less fouling than DTT_COOH_, demonstrating how surface charges from deprotonated carboxyl groups may promote nonspecific adsorption. Considering the HS-MEG-Mix aptasensor (“mix aptasensor”), it had significantly less fouling compared to the other surfaces except for the DTT_COOH_ aptasensor, which has the most excellent antifouling character. The DTT_COOH_ aptasensor may have better antifouling properties compared to the HS-MEG-Mix aptasensor because of more spacing between the tails of the DTT_COOH_ molecules that may facilitate stronger hydration. This can also be due to the higher wettability of a DTT_COOH_ layer compared to an HS-MEG-Mix layer [25].

### 3.3. Sensing of P. aeruginosa in PBS

**Figure 6:**
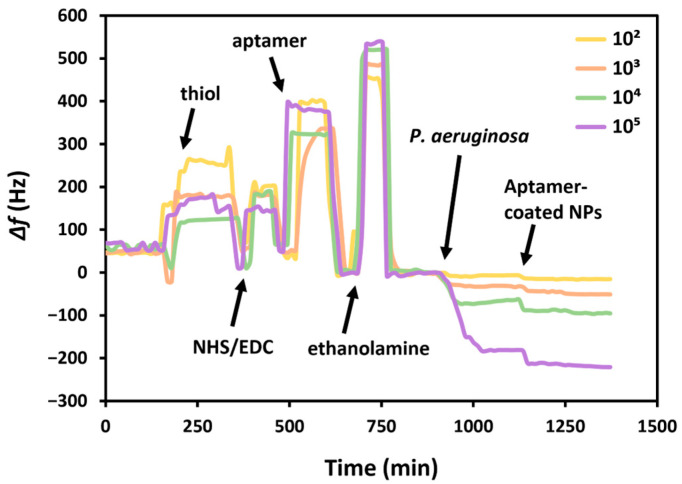
The sensing area, functionalized with HS-MEG-Mix, was further functionalized with HS-MEG-OH thiol in water, NHS/EDC, aptamer, and ethanolamine in flow. Afterwards, different bacterial concentrations were tested, and the frequency varied proportionally with bacterial concentration. The variation in these parameters following exposure to nanoparticles was also proportional with the bacterial concentration.

The constructed aptasensor was used for the detection of bacterial suspensions of *P. aeruginosa* in PBS (Figure 6). The increased frequency during the functionalization of thiol, NHS/EDC, aptamer, and ethanolamine is due to switching between different solutions; shifting the PBS running buffer to other aqueous solutions causes an increased frequency, while returning to PBS subsequently decreases the frequency. The sensor was exposed to different concentrations of bacteria for 20 min, which caused reductions in the resonance frequency, proportional to the concentration of the pathogen. This demonstrated the adsorption of *P. aeruginosa* by its interaction with the aptamer on the crystal surface.

Since the liquid medium used for the bacterial suspension is PBS, i.e., the same as the running buffer, there was no need to do a test without bacteria. Following functionalization with HS-MEG-Mix, aptamer, and ethanolamine, the surface is exposed to different concentrations of bacteria which caused proportional changes in frequency. Furthermore, to increase the sensitivity of the sensor, the suspension of AuNPs functionalized with the same *P. aeruginosa* binding aptamer was then flowed. The result was a further variation in the frequency, increasing as the bacterial concentration increased. This demonstrates that nanoparticles bind specifically to bacteria, as otherwise there would have been an equal frequency variation with all bacterial concentrations.

Table S5.1 in the Supplementary Material highlights the proportionality of the frequency variations due to the increase in *P. aeruginosa* concentrations and subsequent incubation with the nanoparticle suspension. The bacterial concentration is proportional with the variations in frequency, which was calculated considering the difference in the baselines before and after exposure to the bacteria suspension and the nanoparticles.

The frequency variations were calculated considering the average initial frequency of the crystal prior to the exposure of the bacterial suspension or aptamer-coated AuNPs, and the frequency variation immediately after exposure, considering the average frequency in the final 5 min after a wash-off in PBS. Although there is always a drop in frequency, to better display the data, the frequency variation is provided as a positive value.

**Figure 7:**
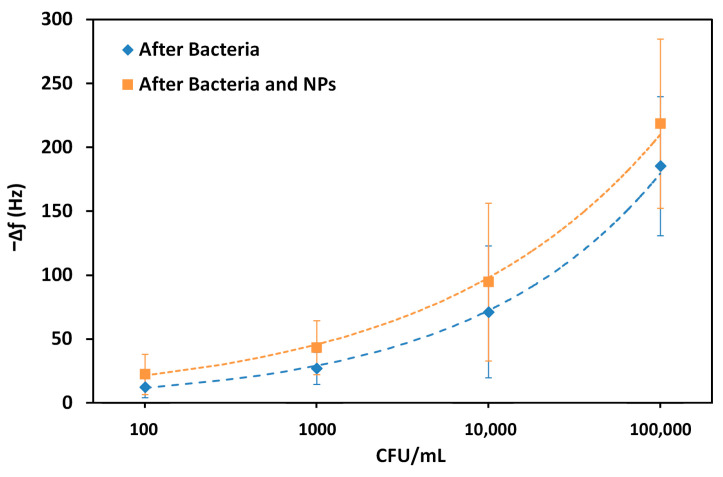
Changes in the resonance frequency of TSM crystals following exposure to increasing concentrations of *P. aeruginosa* (on a logarithmic scale), before the subsequent exposure to specific aptamer-coated AuNPs (blue) and after (orange).

The frequency shifts were used to demonstrate a trend with the logarithm of the bacterial concentration, before and after exposure with the aptamer-coated AuNPs (Figure 7). In this way, the limit of detection (LOD) can subsequently be calculated using the standard deviation of the baseline (S), and the concentration (CFU mL^−^^1^) is calculated at a frequency shift of 3S plus the estimate between the run standard deviation using the calculated trendlines.

The LOD was calculated by the Power based trendline in MS Excel. In this way, a LOD of 86 CFU mL^−^^1^ was obtained, and this value decreased to 68 CFU mL^−^^1^ upon exposure to the AuNPs. The correlation coefficient, R^2^, was also calculated and remained similar, at 0.998 and 0.997, respectively. Although these differences are not large, the values demonstrate that it is possible to increase the sensitivity in the detection of bacteria by incorporating nanoparticles. These limits of detection were extrapolated from the trendlines and were not directly tested.

### 3.4. Sensing of P. aeruginosa in Milk

**Figure 8:**
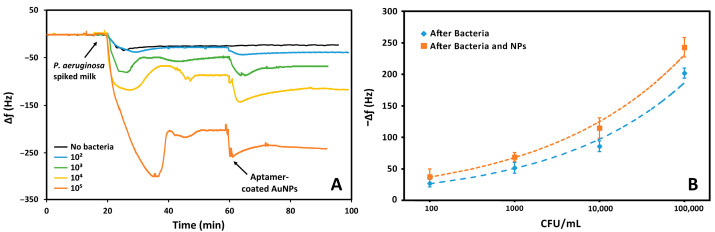
(**A**) The response of the aptasensor to different bacterial concentrations and AuNPs, in which the frequency was proportional to the bacterial concentration. (**B**) The variation in the frequency following exposure to nanoparticles also varied proportionally with the bacterial concentration.

The aptasensor was also employed for the detection of bacterial suspensions of *P. aeruginosa* in milk. The sensing surface was exposed to different concentrations of bacteria, ranging from 10^2^ to 10^5^ CFU, for 20 min. This exposure caused a reduction in the resonance frequency, proportional to the concentration of the pathogen. Since the medium used for this analysis is milk, a test without bacteria was necessary. As Figure 8 shows, upon further functionalization of the sensor the surface is exposed to different concentrations of bacteria which caused proportional changes in frequency. To increase the sensitivity of the sensor, the suspension of AuNPs functionalized with the same *P. aeruginosa* aptamer was then flowed. The result was a further variation in the frequency. Furthermore, the frequency changes due to the binding of AuNPs increased with increasing bacterial concentration. This demonstrates that nanoparticles bind specifically to bacteria.

Table S6.1 in the Supplementary Material highlights the proportionality of the frequency variations due to the increase in *P. aeruginosa* concentrations and subsequent incubation with the nanoparticle suspension. The bacterial concentration is proportional with the variations in frequency that was calculated considering the difference in the baselines before and after exposure to the bacteria suspension and the nanoparticles. The frequency variations were calculated considering the average initial frequency of the crystal prior to the exposure of the bacterial suspension or aptamer-coated AuNPs, and after exposure following a wash-off in PBS.

The frequency shifts were used to demonstrate a trend with the logarithm of the bacterial concentration, before and after exposure with the aptamer-coated AuNPs, as we previously did with the detection in PBS, also considering the frequency shift caused by whole milk with no bacteria present. The limit of detection was calculated by the power based trendline in MS Excel. In this way, a LOD of 157 CFU mL^−^^1^ was obtained, and this value decreased to 46 CFU mL^−^^1^ upon exposure to the AuNPs. The correlation coefficient was also calculated and remained similar, at 0.989 and 0.994, respectively. Interestingly, the system was more sensitive to *P. aeruginosa* in milk following the addition of nanoparticles compared to PBS, despite milk being a more complex medium. As before, these limits were extrapolated from the data.

### 3.5. Specificity of P. aeruginosa Detection

**Figure 9:**
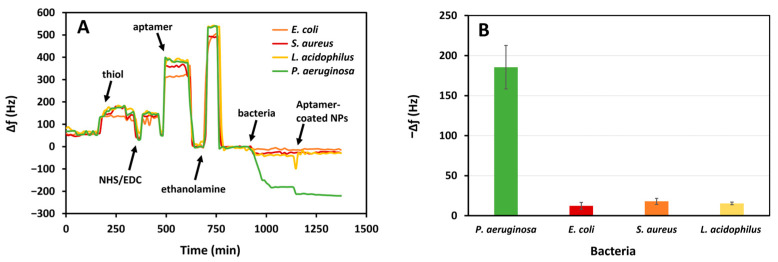
The response of the aptasensor to *P. aeruginosa* compared to *E. coli*, *S. aureus*, and *L. acidophilus* in whole milk (10^5^ CFU mL^−^^1^): (**A**) frequency variations and (**B**) average changes in frequency.

The aptasensor was tested against high concentrations of *S. aureus*, *E. coli*, and *L. acidophilus* (10^5^ CFU mL^−^^1^) in PBS to determine the sensor’s specificity for *P. aeruginosa*. The fast change during the functionalization steps is due to the difference in the density and composition of the solutions. We analyzed the variation in frequency after the PBS baselines. The aptasensor had a significant response to *P. aeruginosa* relative to other bacteria (Figure 9). The average frequency shifts were 185.5 Hz, 12.3 Hz, 17.9 Hz, and 15.3 Hz for *P. aeruginosa*, *E. coli*, *S. aureus,* and *L. acidophilus*, respectively. Therefore, the aptasensor has a specificity of 93.4%, 90.4%, and 91.8% with respect to *E. coli*, *S. aureus,* and *L. acidophilus.* The negligible frequency shift for other bacteria can be attributed to minimal nonspecific events. The greater response to *P. aeruginosa* indicates that the aptasensor has a high specificity for this pathogen relative to the other tested bacteria.

### 3.6. TEM and SEM Microscopy

The gold nanoparticles were analyzed with TEM before and after their functionalization, which showed that the nanoparticles are uniform in size (S7.1 in the Supplementary Material). However, it is difficult to appreciate the differences after functionalization, since the aptamers are very small.

**Figure 10:**
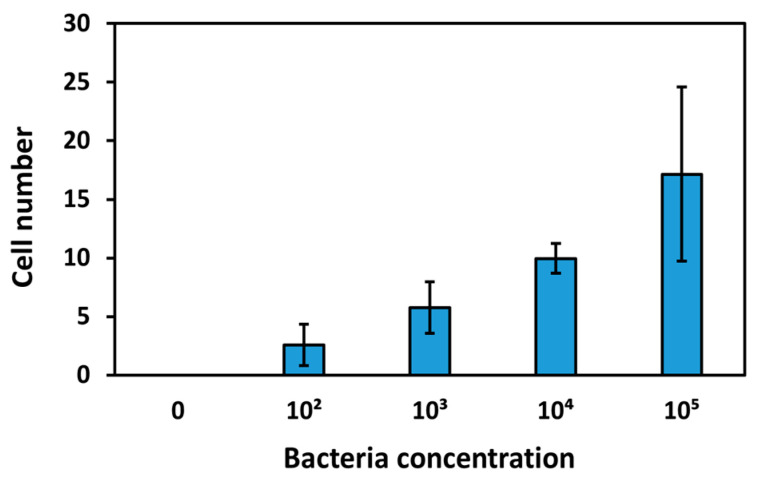
The average number of counted cells as a function of the concentration of bacteria after the experiments of detection. The number of cells is an average of the bacterial count from twenty SEM images. The error bars were determined from the standard deviation of the twenty SEM measurements.

After performing measurements of the sensing surfaces of the TSM crystals with different bacterial concentrations in milk, these were analyzed with SEM. Twenty photographs were taken for each concentration, using the same parameters, and the bacterial cells were counted. Selected images are shown in S7.2 in the Supplementary Material. From the analyses performed, the average number of cells increases with increasing bacterial concentration, demonstrating that the bacteria are effectively immobilized on the surface (Figure 10).

**Figure 11:**
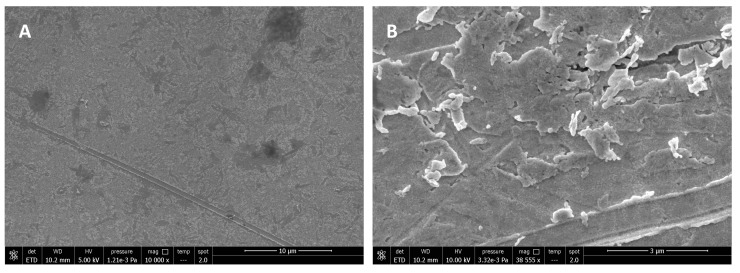
SEM images after exposing (**A**) antifouling and (**B**) bare electrode surfaces to milk. The antifouling surface in (**A**) is the aptasensor, comprising HS-MEG-Mix thiols, aptamer, and HS-MEG-COOH elongated with ethanolamine.

Analyses were also conducted on surfaces used for experiments without bacteria, and the absence of cells in the images can confirm that the investigation was carried out without contaminating the samples with other bacterial cells. Analyses were performed on TSM crystal surfaces not functionalized with the antifouling SAM, and following experiments conducted with milk as a fouling agent. It is interesting to note that layers of casein and micelles can be clearly seen on bare surfaces, while discs modified with the antifouling layer do not have protein deposits (Figure 11). Instead, some salts from the PBS running buffer can be seen on the antifouling surfaces since the discs were not rinsed with Milli-Q water after removal from the TSM instrument for microscopic imaging.

### 3.7. Literature Comparison

A literature search was conducted on the development of aptamer-based biosensors capable of detecting *P. aeruginosa* in recent years (2019–2024), and Table 1 shows these studies, highlighting some aspects. There is a shortage of aptasensors based on piezoelectric transduction, while those based on electrochemical transduction are the predominant ones. Some works appear to be very promising, as they reach a very low detection limit. However, these sensors are often complex, incorporating labels or nanostructures that require fairly complicated sensor preparation and measurement. Furthermore, many of these sensors have not been tested in real samples, or their limit of detection has not been determined.

## 4. Conclusions

This work focused on an aptasensor for detecting *P. aeruginosa*, which was used to quantify the bacterium in whole milk samples. The aptasensor’s self-assembled thiol layer, made up of the antifouling molecules HS-MEG-COOH and HS-MEG-OH (HS-MEG-Mix), bound with the aptamer and passivated with ethanolamine, provided the most antifouling against whole milk. Quantitative detection of *P. aeruginosa* was achieved in PBS using the thickness shear mode acoustic aptasensor. The sensor has high selectivity for *P. aeruginosa* as negligible signals were measured for *E. coli*, *S. aureus*, and *L. acidophilus*. The sensitivity of the aptasensor was improved by incorporating AuNPs after bacterial detection, which were coated with *P. aeruginosa*-specific aptamers. The nanoparticles bound to the immobilized bacteria on the mass-sensitive surface, causing a slight decrease in the resonance frequency of the crystal in proportion to bacterial concentration. The nanoparticles improved the extrapolated limits of detection from 86 to 68 CFU mL^−1^ in PBS and 157 to 46 CFU mL^−1^ in milk due to their small diameter of 5 nm and possibly because of the working concentration of 0.5 O.D. Additionally, imaging the surface with SEM following the binding of different concentrations of bacteria to the aptasensor showed a clear correlation between the concentration of bacteria in solution and the number seen on the surface. SEM also demonstrated the effectiveness of the aptasensor’s antifouling behavior compared to the bare surface, since milk components were not deposited on the antifouling surface. Detecting *P. aeruginosa* with the aptasensor in conjunction with nanoparticles is a proof-of-concept in our work; future studies will investigate how nanoparticles with larger diameters at various concentrations can further improve the limit of detection. In addition, as the aptasensor was demonstrated to be antifouling in whole milk, further studies can be performed to test the ability of the aptasensor to detect *P. aeruginosa* without the pretreatment of whole milk samples.

## Figures and Tables

**Table 1:** A review of selected works on aptasensors used for the detection of *P. aeruginosa* from 2019–2024.

Material Platform	Method of Detection	LOD (CFU mL^−1^)	Linear Range (CFU mL^−1^)	Sample	Reference
*Acoustic or magnetic aptasensors*					
Polyadenylated-DNA	IDE-MSPQC	9	81–8.1 × 10^5^	Buffer	[15]
Polyadenylated-DNA	IDE-MSPQC	52	1.9 × 10^2^–10^6^	Simulated blood	[15]
Aptamer-coated MNPs	LF-MRI	100	3.1 × 10^2^–3.1 × 10^7^	PBS buffer	[26]
Aptamer/Aptamer-coated AuNPs	TSM	68/46	10^2^–10^5^	PBS/Milk	This work
*Electrochemical aptasensors*					
AuNPs	Peroxidase-like activity	60	60–6 × 10^7^	Water	[27]
AgNPs/c-g-C3N4/Apt	MWCNT, DPV	1.0	10–10^7^	PBS buffer	[28]
Chitosan-GCE	EIS, CV	3.0	10–10^7^	PBS buffer	[29]
AgNPs-Electrodeposited GCE	EIS, CV	33.0	10^2^–10^7^	PB buffer	[30]
Aptamer-MIP/AuNPs GCE	EIS, CV	1.0	10–107	PB buffer	[31]
Aptamer/DNA tetrahedron	EIS	5.0	4.4 × 10^1^–10^5^	Piped water, diluted urine, and PBS	[32]
*Optical aptasensors*					
Aptamer-coated AuNPs	Colorimetric	10^5^ (naked eye), 10^4^ (UV-vis)	10^5^–10^8^	BHI medium	[33]
(PDA-PEI) copolymer dots	Fluorescence	1.0	10–10^7^	TRIS buffer	[17]
Aptamer/DNA tetrahedron	Fluorescence	14.0	4.4 × 10^2^–10^9^	Piped water, diluted urine, and PBS	[32]
Aptamer-DTNPs, hemin	Colorimetric/electrochemical	1.0	1–10^7^	Buffer	[34]

AgNPs/c-g-C_3_N_4_/Apt: aptamer immobilized on graphitic carbon nitride complex with silver nanoparticles; CV: cyclic voltammetry; DPV: differential pulse voltammetry; DTNPs: DNA tetrahedral nanoprobes; EIS: electrochemical impedance spectroscopy; GCE: glassy carbon electrode; IDE: interdigital electrode; LF-MRI: low-field magnetic resonance imaging; MIP: molecularly imprinted polymers; MNPs: magnetic nanoparticles; MSPQC: multi-channel series piezoelectric quartz crystal; MWCNT: multi-walled carbon nanotubes; PDA-PEI: polydopamine-polyethyleneimine.

## Data Availability

Data are contained within the article and the Supplementary Material.

## Supplementary Materials

The following supporting information can be downloaded at https://www.mdpi.com/article/10.3390/bios15030146/s1. S1: Secondary Structure of DNA Aptamer. The secondary structure of the aptamer (5′-CCC CCG TTG CTT TCG CTT TTC CTT TCG CTT TTG TTC GTT TCG TCC CTG CTT CCT TTC TTG-3’) was analyzed using the OligoAnalyzer ToolTM (eu.idtdna.com/pages/tools/oligoanalyzer, accessed on 18 February 2025) software. Nine variations in the structure were found for the aptamer, where three are characterized with the greatest stability due to low Gibbs free energy: (A) −0.69 kcal/mol, (B) −0.41 kcal/mol, and (C) −0.38 kcal/mol are presented in Figure S1. Figure S1. Secondary structures of the aptamer selective for *P. aeruginosa* predicted by the OligoAnalyzer Tool^TM^. S2: Bacteria Preparation. Lysogeny broth (LB) was used to grow PAO1 bacteria at 37 °C overnight. The grown solution was serially diluted from 1/2 to 1/1,000,000,000 times in LB. Each solution was spotted onto agar plates (10 μL × 3), as well as measured using a UV-1600PC spectrometer (VWR International, Mississauga, ON, Canada) to measure the optical density at 600 nm (OD600). The plates were incubated overnight at 37 °C and spot counted to calculate CFU per OD600. Figure S2. CFU/mL concentration of PAO1 in LB compared to their optical density at 600 nm. A linear relationship between the OD600 measurements and CFU counts for PAO1 in LB was found: CFU/mL = 1 × 10^9^ *x* + 3 × 10^7^, where *x* is the OD600 value. Using this relationship, the grown solutions of PAO1 could be diluted to the desired concentration for measurement. S3: Nanoparticle Characterization. The analysis of correct functionalization of AuNPs was carried out by a UV-Vis spectrophotometer. In the Figure S3, we can see that two peaks were detected: the first peak, around 519 nm, relates to the absorption of the 5 nm diameter gold nanoparticles. This value was also used to control the nanoparticle concentration, to maintain the same AuNPs concentration for each *P. aeruginosa* detection experiment at different concentrations (CFU mL^−1^). The second peak ~260 nm relates to the absorption of nucleic acids of the aptamer. The bond between the aptamer and the mixed SAM, HS-MEG-Mix, is believed to have been covalent for two main reasons: (1) the same procedure was performed as used for the functionalization of the TSM gold electrodes, and (2) the mixed SAM has hydrophilic and antifouling characteristics which should precisely prevent non-specific interactions with biological material. Figure S3. Characterization of aptamer-functionalized gold nanoparticles by UV-Vis. Two characteristic peaks of DNA and nanoparticle absorption can be noted. S4: Milk Antifouling Test with TSM. Table S4.1. The changes in frequency, Δ*f*, after whole milk exposure to surfaces coated with HS-MEG-COOH, HS-MEG-OH, thiol mix, and the aptasensor (further modified with aptamer and ethanolamine). S5: Sensing of *Pseudomonas aeruginosa* in PBS. Table S5.1. The frequency and dissipation changes following bacteria incubation and the overall variations after the aptamer-coated nanoparticles. S6: Sensing of *Pseudomonas aeruginosa* in Milk. Table S6.1. The frequency changes following incubation of bacteria in milk and the overall variations after the aptamer-coated nanoparticles. S7: TEM and SEM Microscopy Results. S7.1: TEM images. Figures: Before functionalization, After functionalization. S7.2. SEM images. Figures: 0 CFU mL^−1^, 10^2^ CFU mL^−1^, 10^3^ CFU mL^−1^, 10^4^ CFU mL^−1^10^5^ CFU mL^−1^, bare electrode surface with milk deposits.
