# Supplementary material for: Nanoparticle-Enhanced Acoustic Wave Biosensor Detection of Pseudomonas aeruginosa in Food
Source: Biosensors (Basel). 2025 Feb 25;15(3):146. doi: 10.3390/bios15030146 (PMC11940165; doi:10.3390/bios15030146)

Supplementary Material

Nanoparticle-Enhanced Acoustic Wave Biosensor Detection of *Pseudomonas Aeruginosa* in Food

Sandro Spagnolo,[1] Katharina Davoudian,[2] Brian De La Franier,[2] Robert Kocsis,[3] Tibor Hianik,[1] and Michael Thompson[2]

[1] Faculty of Mathematics, Physics and Informatics, Comenius University, Mlynská dolina F1, 84248 Bratislava, Slovakia; sandrospagnolo1@gmail.com (S.S); tibor.hianik@fmph.uniba.sk (T.H.)

[2] Department of Chemistry, University of Toronto, 80 St. George Street, Toronto, ON M5S 3H6, Canada; k.davoudian@mail.utoronto.ca (K.D.); brian.delafranier@mail.utoronto.ca (B.D.L.F.); edmund.chan@mail.utoronto.ca (E.C.)

[3] Hungarian Dairy Research Institute Ltd., 1 József Csiszár Street, Mosonmagyaróvár, 9200, Hungary; rkocsis@mtki.hu (R.K)

* Correspondence: m.thompson@utoronto.ca (M.T.)

S1. Secondary Structure of the DNA Aptamer

The secondary structure of the aptamer (5'-CCC CCG TTG CTT TCG CTT TTC CTT TCG CTT TTG TTC GTT TCG TCC CTG CTT CCT TTC TTG-3') was analyzed using the OligoAnalyzer Tool™ (eu.idtdna.com/pages/tools/oligoanalyzer) software. Nine variations in the structure were found for the aptamer, where three are characterized with the greatest stability due to low Gibbs free energy: (A) -0.69 kcal/mol, (B) -0.41 kcal/mol, and (C) -0.38 kcal/mol are presented in **Figure S1**.

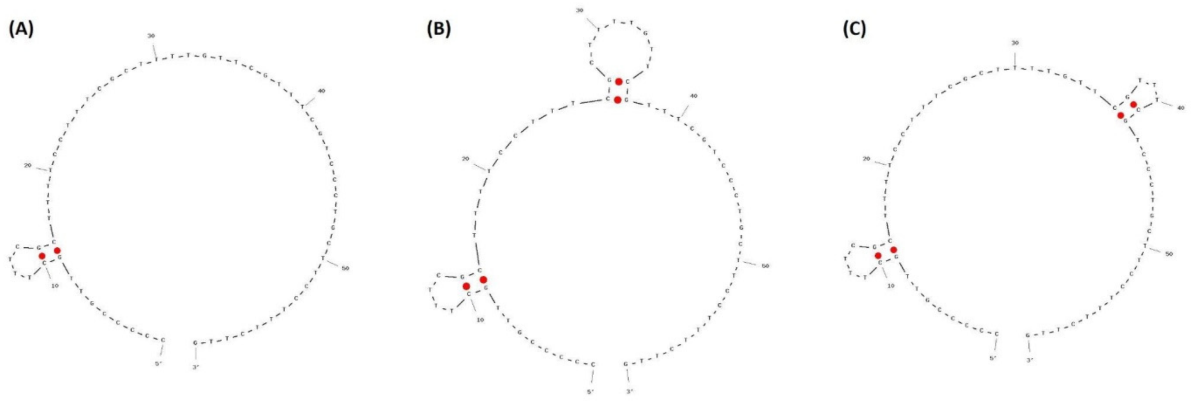

Figure S1. Secondary structures of the aptamer selective for *P. aeruginosa* predicted by the OligoAnalyzer Tool™. The secondary structures correspond to different Gibbs free energy: (A) -0.69 kcal/mol, (B) -0.41 kcal/mol, and (C) -0.38 kcal/mol.

S2. Bacteria Preparation

Lysogeny broth (LB) was used to grow PAO1 bacteria at 37 °C overnight. The grown solution was serially diluted from 1/2 to 1/1,000,000,000 times in LB. Each solution was spotted onto agar plates (10 μL × 3), as well as measured using a UV-1600PC spectrometer (VWR International, Mississauga, Canada) to measure the optical density at 600 nm (OD600). The plates were incubated overnight at 37 °C and spot counted to calculate CFU per OD600.

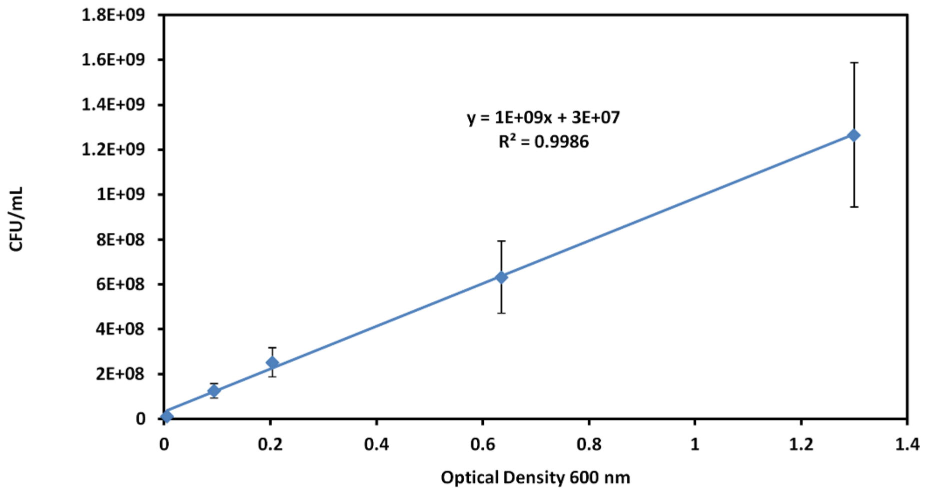

Figure S2. CFU/mL concentration of PAO1 in LB compared to their optical density at 600 nm.

A linear relationship between the OD600 measurements and CFU counts for PAO1 in LB was found:

$$\frac{CFU}{mL} = 1 \times 10^9 x + 3 \times 10^7,$$

where x is the OD600 value. Using this relationship, the grown solutions of PAO1 could be diluted to the desired concentration for measurement.

S3. Nanoparticle Characterization

The analysis of correct functionalization of AuNPs was carried out by a UV-Vis spectrophotometer. In Figure S3, we can see that two peaks were detected: the first peak, around 519 nm, relates to the absorption of the 5 nm diameter gold nanoparticles. This value was also used to control the nanoparticle concentration, to maintain the same AuNPs concentration for each *P. aeruginosa* detection experiment at different concentrations (CFU mL^{-1}). The second peak ~260 nm relates to the absorption of nucleic acids of the aptamer.

The bond between the aptamer and the mixed SAM, HS-MEG-Mix, is believed to have been covalent for two main reasons: (1) the same procedure was performed as used for the functionalization of the TSM gold electrodes, and (2) the mixed SAM has hydrophilic and antifouling characteristics which should precisely prevent non-specific interactions with biological material.

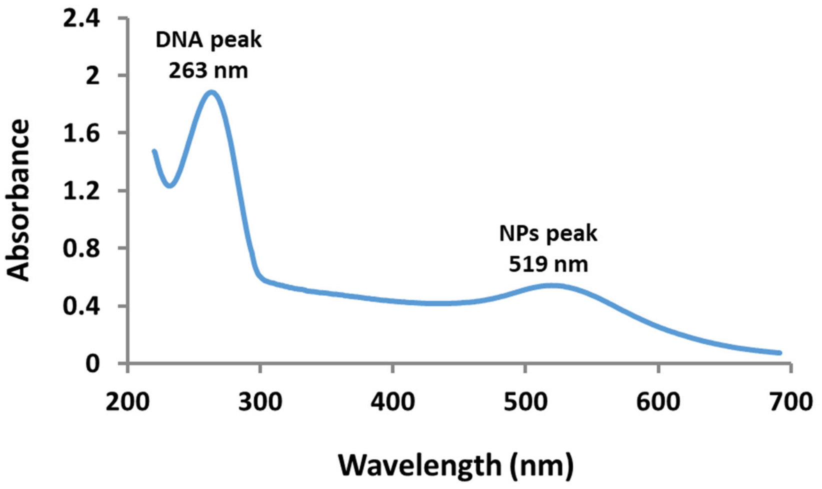

Figure S3. Characterization of aptamer-functionalized gold nanoparticles by UV-Vis. Two characteristic peaks of DNA and nanoparticle absorption can be noted.

S4. Milk Antifouling Test with TSM

Table S4.1. The changes in frequency, Δf, after whole milk exposure to surfaces coated with HS-MEG-COOH, HS-MEG-OH, thiol mix, and the aptasensor (further modified with aptamer and ethanolamine).

SAM	Δf (Hz)
**HS-MEG-COOH**	92.0 ± 14.3
**HS-MEG-OH**	50.0 ± 15.0
**HS-MEG-Mix**	80.9 ± 0.6
**Aptasensor**	17.7 ± 9.4

S5. Sensing of *Pseudomonas aeruginosa* in PBS

Table S5.1. The frequency and dissipation changes following bacteria incubation and the overall variations after the aptamer-coated nanoparticles.

Frequency variations (Hz)

Bacteria (CFU mL^{-1})	-(Δf)$_{bacteria}$	-(Δf)$_{AuNPs}$	-(Δf_{AuNPs} - $\Delta f_{bacteria}$)
10^2	12.4 ± 8.1	22.4 ± 15.7	10.0 ± 7.7
10^3	27.2 ± 12.6	43.5 ± 21.1	16.3 ± 8.5
10^4	71.4 ± 51.6	94.7 ± 61.7	23.3 ± 10.1
10^5	185.5 ± 54.4	218.7 ± 66.2	33.1 ± 11.8

S6. Sensing of *Pseudomonas aeruginosa* in Milk

Table S6.1. The frequency changes following incubation of bacteria in milk and the overall variations after the aptamer-coated nanoparticles.

Frequency variations (Hz)			
Bacteria (CFU mL^{-1})	-(Δf)$_{bacteria}$	-(Δf)$_{AuNPs}$	-(Δf_{AuNPs} - $\Delta f_{bacteria}$)
10^2	27.2 ± 5.8	37.6 ± 12.0	10.4 ± 6.2
10^3	51.6 ± 8.5	69.0 ± 6.8	17.4 ± 5.7
10^4	85.8 ± 8.6	115.1 ± 16.2	29.2 ± 7.6
10^5	201.8 ± 8.1	242.9 ± 15.3	41.1 ± 7.3

S7. TEM and SEM Microscopy Results

S7.1. TEM images

Before functionalization

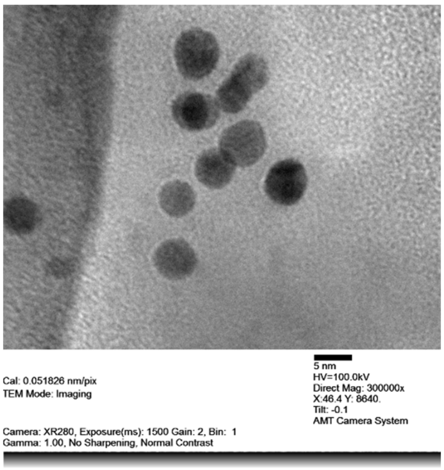

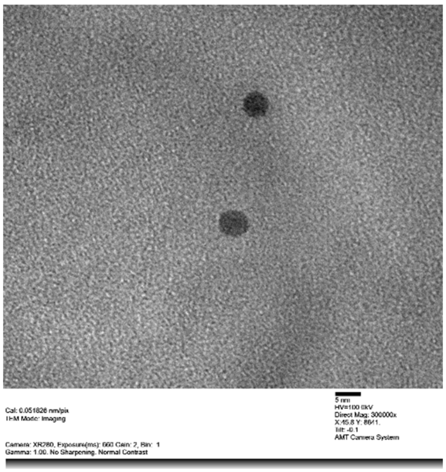

After functionalization

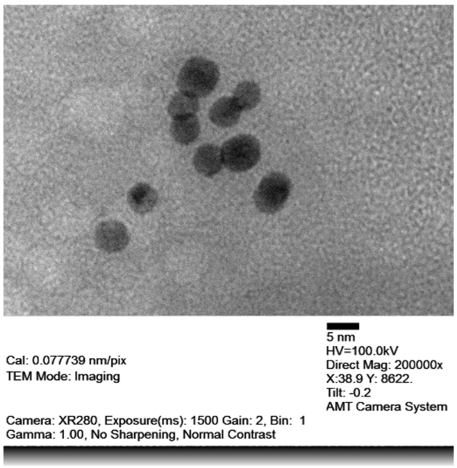

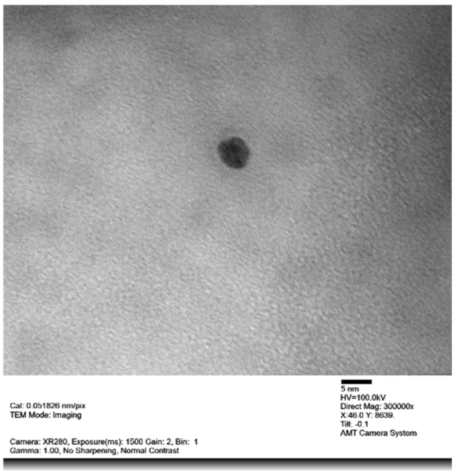

S7.2. SEM images

0 CFU mL^{-1}

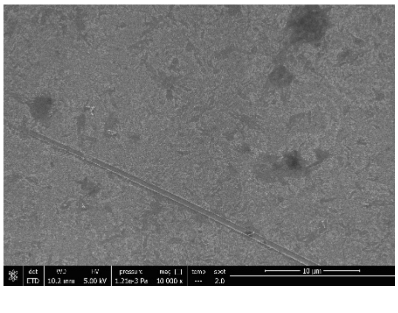

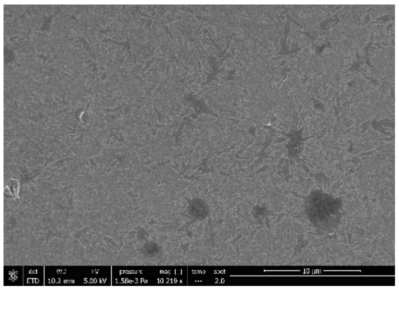

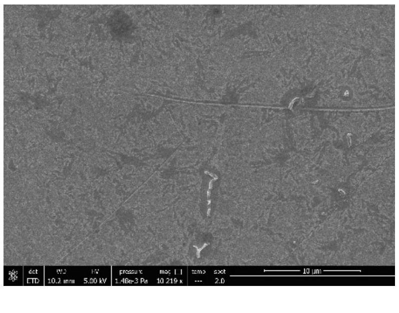

10^2 CFU mL^{-1}

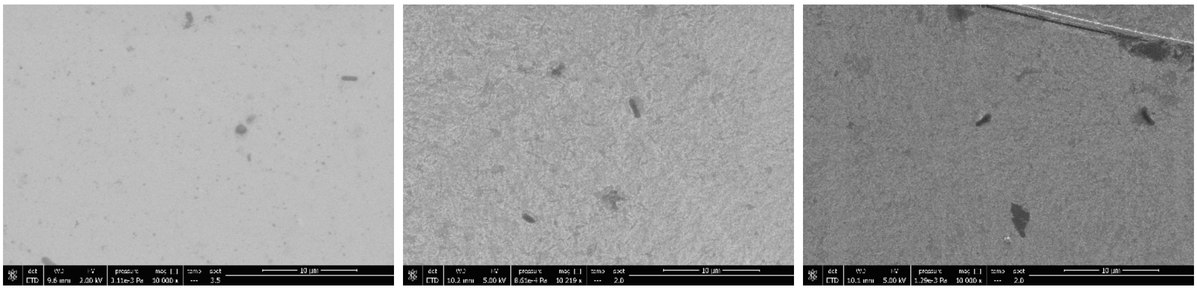

10^3 CFU mL^{-1}

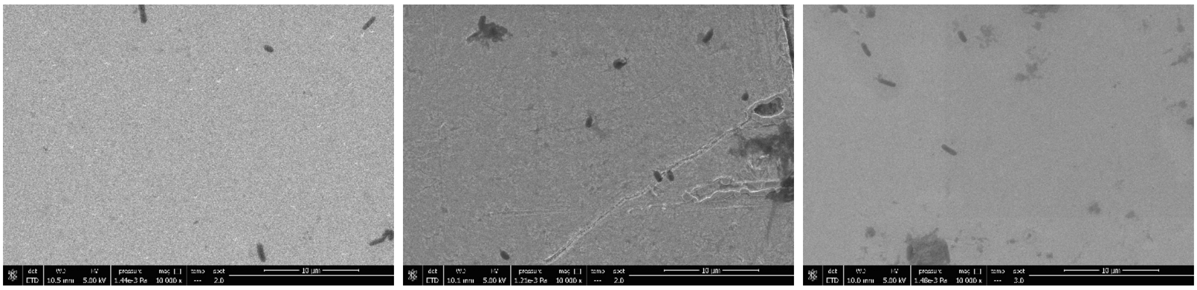

10^4 CFU mL^{-1}

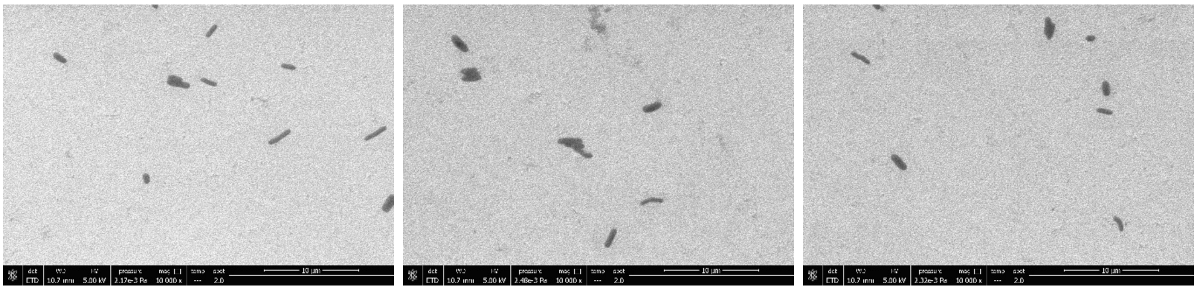

10^5 CFU mL^{-1}

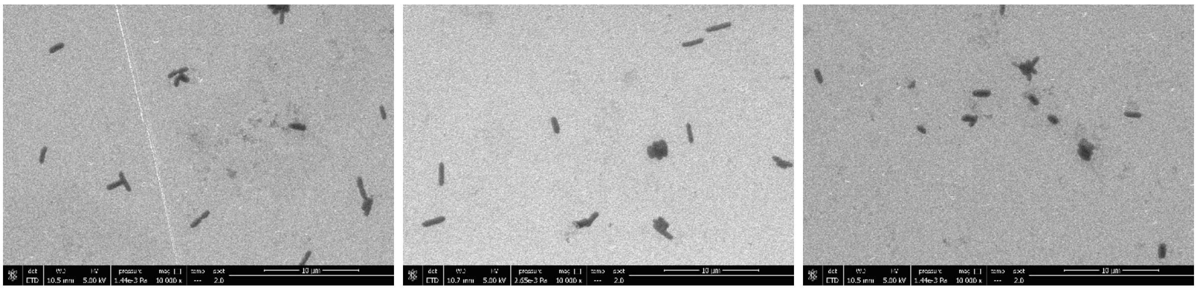

Bare electrode surface with milk deposits

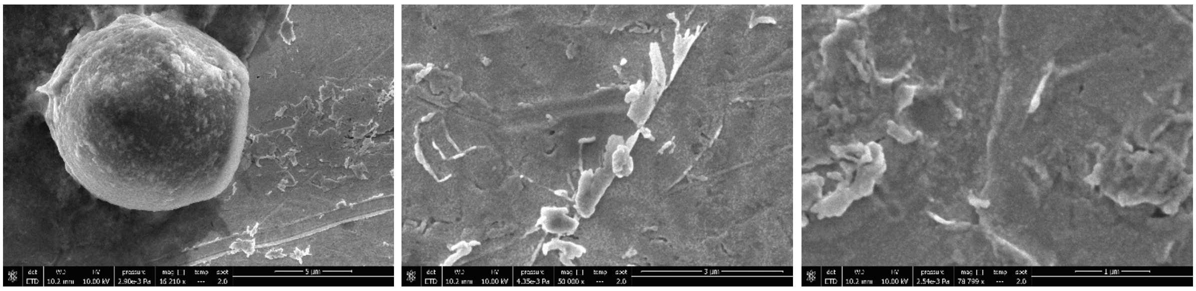

Supplement: Supplementary file 1 [file biosensors-15-00146-s001.zip › biosensors-3455087-supplementary.pdf]
